# Childhood cancer research in oxford III: The work of CCRG on ionising radiation

**DOI:** 10.1038/s41416-018-0182-y

**Published:** 2018-08-21

**Authors:** Gerald M. Kendall, John F. Bithell, Kathryn J. Bunch, Gerald J. Draper, Mary E. Kroll, Michael F. G. Murphy, Charles A. Stiller, Tim J. Vincent

**Affiliations:** 1 Cancer Epidemiology Unit, Nuffield Department of Population Health, University of Oxford, Richard Doll Building, Old Road Campus, Oxford, OX3 7LF UK; 2 Department of Statistics, University of Oxford, 24-29 St Giles’, Oxford, OX1 3LB UK; 3 National Perinatal Epidemiology Unit, Nuffield Department of Population Health, University of Oxford, Richard Doll Building, Old Road Campus, Oxford, OX3 7LF UK; 4 Formerly of Childhood Cancer Research Group, University of Oxford, Oxford, UK; 5 Nuffield Department of Women’s and Reproductive Health John Radcliffe Hospital, University of Oxford, Oxford, OX3 9DU UK; 6 National Cancer Registration and Analysis Service, Public Health England, Chancellor Court, Oxford Business Park South, Oxford, OX4 2GX UK

**Keywords:** Risk factors, Oncology

## Abstract

**Background:**

High doses of ionising radiation are a known cause of childhood cancer and great public and professional interest attaches to possible links between childhood cancer and lower doses, particularly of man-made radiation. This paper describes work done by the Childhood Cancer Research Group (CCRG) on this topic

**Methods:**

Most UK investigations have made use of the National Registry of Childhood Tumours and associated controls. Epidemiological investigations have included national incidence and mortality analyses, geographical investigations, record linkage and case-control studies. Dosimetric studies use biokinetic and dosimetric modelling.

**Results:**

This paper reviews the work of the CCRG on the association between exposure to ionising radiation and childhood cancer, 1975–2014.

**Conclusion:**

The work of CCRG has been influential in developing understanding of the causes of 'clusters' of childhood cancer and the risks arising from exposure to ionising radiation both natural and man-made. Some clusters around nuclear installations have certainly been observed, but ionising radiation does not seem to be a plausible cause. The group’s work has also been instrumental in discounting the hypothesis that paternal preconception irradiation was a cause of childhood cancers and has demonstrated an increased leukaemia risk for children exposed to higher levels of natural gamma-ray radiation.

## Introduction

This paper is one of three describing the Oxford Survey of Childhood Cancers (OSCC) and the work of the Childhood Cancer Research Group (CCRG), which was developed from the OSCC. The first paper describes the history of the OSCC under the direction of Alice Stewart^1^. The second paper describes the foundation of the CCRG and its work on topics other than ionising radiation^2^. This paper complements Draper et al. by summarising the work of the CCRG on ionising radiation.

High doses of ionising radiation are a known cause of childhood cancer, taken here as cancers arising before the 15th birthday^3^. The evidence is particularly clear regarding leukaemia and thyroid cancer^3^. Nevertheless, evidence, some of it from the OSCC or NRCT, suggests that other cancers may also be induced^4,5^. In this paper we refer to 'high' and 'low' doses of radiation. The definition of these terms is a thorny one^6^. Here we are generally thinking of low doses as around the level of natural background radiation, a few mSv per year^7^. We mention high doses of radiation in the effects of radiotherapy and low doses in the context of natural background radiation and diagnostic medical exposures. Much of the discussion involves childhood cancers around nuclear installations where effects have been postulated at doses below those at which observable effects would normally be expected.

## Materials and Methods

Most of the work of the CCRG was based on the National Registry of Childhood Tumours (NRCT), a comprehensive register of cases of childhood cancers diagnosed before the fifteenth birthday in Great Britain (England, Wales and Scotland). Controls, matched on age, sex and birth registry were later added to the NRCT and have greatly increased its utility. A fuller description of the NRCT is given by Draper et al.^2^.

Research conducted by CCRG using the NRCT has been very wide-ranging, covering descriptive studies of trends in incidence and survival of childhood cancer and also studies of possible aetiological factors. The aetiological studies encompassed both ionising and non-ionising radiation and also non-radiation causes. Draper et al.^2^ summarise the latter and this paper the former. Details of the data underlying and statistical methods employed in the different studies can be found in the referenced publications

## Results

### Clusters of childhood cancer and environmental risks around nuclear installations

#### Clusters of childhood cancer

In November 1983 a programme 'Windscale—The Nuclear Laundry' was shown on British television. It claimed that there was a substantial excess incidence of cancer in children and young people in the area around what is now called the Sellafield nuclear plant (originally called 'Windscale and Calder Works'). In particular, it claimed a ten-fold excess of leukaemia among children aged under 10 years in the nearby village of Seascale. There was wide-spread and deep public concern, especially amongst those living in the area, particularly in Seascale. It was suggested that excess childhood cancer might be a consequence of emissions from the Sellafield plant, which released radioactivity into the environment in normal operation but which had also generated some accidental releases^8^, most notably during the Windscale Fire of 1957^9^.

The UK government set up an expert Independent Advisory Group (the 'Black Committee') to investigate the claims. It worked with astonishing speed by the current standards of such bodies and published its report within a year^10^. The Black report confirmed that there did appear to be high levels of leukaemia in those aged below 25 years. However, the estimated radiation doses to the local population were too low to account for the observed leukaemia incidence. The Black Committee recommended additional scientific investigations of the apparent excess and of the radiation doses incurred by members of the public in the area.

In November 1985, in response to the recommendations of the Black Committee, the UK Government set up the Committee On Medical Aspects of Radiation in the Environment (COMARE). COMARE has published a number of reports, most of which are relevant to the general area of childhood cancer clusters around nuclear installations. The work of COMARE, as of the Black Committee before it, was supported by a number of other organisations, in particular the CCRG and the National Radiological Protection Board (NRPB, later part of the Health Protection Agency and now within Public Health England). The Table 1 shows the main COMARE publications to which CCRG contributed.

**Table 1:** COMARE Publications on Ionising Radiation with significant CCRG involvement

Report	Topic	Year
1	Implications of new data on Sellafield releases on the incidence of Cancer in West Cumbria	1986
2	Incidence of leukaemia in young people near Dounreay Nuclear Establishment Caithness Scotland.	1988
3	Cancer incidence around AWE Aldermaston and ROF Burghfield	1989
4	Childhood cancer incidence in the vicinity of Sellafield	1996
5	Cancer incidence around Greenham Common Airbase	1998
6	Review of radioactive particles around the Dounreay nuclear site	1999
7	Childhood cancer following preconceptional radiation exposure	2002
8	Effect of preconceptional radiation exposure on pregnancy	2004
9	Advice on CERRIE’s review of internal radiation emitters risks	2004
10	Childhood cancer incidence around UK nuclear sites	2005
11	Childhood cancer distribution in Great Britain 1969 to 1993	2006
14	Childhood leukaemia incidence around UK nuclear power plants	2011
15	Radium contamination in the area around Dalgety Bay	2014
17	Further consideration of the incidence of cancers around the nuclear installations at Sellafield and Dounreay	2016

Not long after the publication of the conclusions of the Black Committee, a paper was published reporting an excess of childhood leukaemia in the vicinity of the Dounreay nuclear plant in Northern Scotland^11^. Dounreay is the other plant in Britain where nuclear fuel is reprocessed, and inevitably it was suggested that there was a causal link between some aspect of the activities at the two sites and childhood cancer in surrounding areas.

Concern did not stop at nuclear reprocessing plants and studies were carried out to investigate rates of childhood and other cancers around all kinds of nuclear installations, including nuclear power plants (NPP), all over the world. Taken altogether, the literature on cancer rates around nuclear installations is formidable. A 2008 review by^12^, restricted to childhood leukaemia, collected several hundred publications. Care is needed in the interpretation of these studies. There can be considerable variation in exactly what is being considered in, for example, age range, geographical location and diagnostic grouping. Leukaemia and non-Hodgkin lymphoma (LNHL) are often considered together because it was considered plausible that in the early years the differential diagnosis was not necessarily consistent; indeed in reviewing these diagnoses COMARE found that three cases of childhood NHL should be more appropriately registered as ALL.^13^.

An apparently noteworthy excess can sometimes be a result of choosing the parameters so as to include the maximum number of cases, but as few other individuals as possible (the ''Texas Sharpshooter Fallacy'')^14^. Glass et al.^15^ give a nice example of such gerrymandering. A further caveat in the interpretation of these studies is that many nuclear facilities have been investigated and thus chance findings of excesses are to be expected—and publication bias is also likely. Nevertheless, some cancer clusters near nuclear installations certainly exist.

#### Environmental risks around nuclear installations

It quickly became apparent that natural radiation was responsible, as it is generally, for a large proportion (about 80%) of the radiation dose to a typical inhabitant of the Sellafield area^10,16–18^. Since doses from Sellafield discharges were much smaller than those from natural sources it was implausible that they could cause a large local increase in LNHL.

In 1993, Draper et al.^19^ published a study of cancer over the period 1963–90 in the area around Sellafield. The age range considered was 0–74, but with especial attention to ages 0–24. Data from the NRCT were used for cancer cases aged under 15 years. Rates of incidence of cancer were calculated using data from population based cancer registries and from special surveys. Draper et al. confirmed the increased childhood leukaemia/LNHL incidence at ages 0–24 y in Seascale between 1963 and 1983. For the first time they demonstrated that an increased risk continued beyond this period to 1984–90. These increases were relative to national rates or to the surrounding areas. For the immediately surrounding areas—that is, the county districts (CDs) of Allerdale and Copeland excluding Seascale and in the remainder of Cumbria—there was no evidence of an increased incidence of cancer among those aged 0–24 years in either period. Draper et al. concluded 'The increased risk is unlikely to be due to chance but the reasons for it are .

In 1994 Bithell et al.^20^ published the first comprehensive surveys of LNHL incidence in children below age 15 around all nuclear installations (both NPP and other nuclear installations such as reprocessing, research and defence establishments) in England and Wales. The study considered electoral wards within 25 km of 23 nuclear installations and six control sites that had been investigated for suitability for power plants but never used. Observed and expected numbers of cases were compared and distance trend tests designed to be sensitive to excess incidence in close proximity to a putative source of risk were calculated. Bithell et al. found little evidence of elevated incidence in the vicinity of NPP. Evidence for clusters around other nuclear installations was stronger, in particular for Sellafield where the effect was entirely due to Seascale village. These analyses were later updated for the whole of Britain for the 2005 COMARE Tenth Report^21^.

The 1994 study by Bithell et al of LNHL to age 15 around nuclear installations in England and Wales^20^ had used age and disease classifications consistent with British practice. Meanwhile and during the ensuing decades, studies of childhood leukaemia or cancer incidence around nuclear installations were set up in several other countries, notably France^22^, Germany^23^ and Switzerland^24^. The most influential of these was the German study of childhood cancer in the areas around NPP (KiKK, 'Kinderkrebs in der Umgebung von Kernkraftwerken')^23^. This was a study of 593 leukaemia cases aged up to 5 y and 1766 matched controls. The investigators found a statistically significant odds ratio of 2.19 (lower 95% one-sided confidence limit 1.51) for residential proximity within 5 km of a nuclear power plant compared to residence outside this area. They noted that this observation could not be explained under present knowledge of radiation protection. The finding of Kaatsch et al. was not entirely due to the known Krümmel cluster of childhood leukaemia^25^.

In 2008 Bithell and co-workers published as close a parallel to KiKK as could be achieved within the constraints of an ecological study, i.e. using smaller regions around the installations^26,27^; specifically, they examined only children with leukaemia under 5 y within 5 km of a plant. The incidence ratio (IR) for cases of acute leukaemia aged under 5 y within 5 km of a nuclear power plant was not significant: *O* = 20, IR = 1.36 (0.83–2.10). Nor were risk coefficients for proximity in the regression analysis significant. Similar analyses and results were reported in COMARE’s 14th report^28^, which included a comparative review of KiKK.

A yet closer parallel with KiKK was published by Bithell et al. in 2013^29^. This was the first British case-control study of the issue, involving 9821 cases of LNHL at ages under 5 y around British NPP. The calendar period covered was between 1962 and 2007. No increased risk associated with residential proximity to NPP was found. Moreover, the risk estimates were incompatible with the German study. It should be noted that people in Britain tend to live further away from NPP than their counterparts in Germany; the percentage of controls living within 5 km being 0.1% and 3.2%, respectively.

In 2014 Bunch et al.^30^ updated the analyses of childhood cancer at ages 0–24 years around Sellafield and Dounreay. The study period was extended from 1963–90 to 2006. Cancer incidence rates, both overall and for diagnostic sub-types, were compared to general population rates. The results for 1963–1990 were consistent with earlier studies. However, there was no excess of cancers around Sellafield (including within Seascale Ward) or Dounreay over more recent years (1991–2006). Bunch et al. also examined all age cancer incidence in those born around either site (but not necessarily living there in later life) and found no increased risk.

#### Infective mechanisms and clusters of childhood leukaemia

There has been increasing interest in recent decades in the possibility that infective mechanisms are important in the aetiology of childhood cancers, particularly leukaemia. CCRG has been involved in a number of these investigations. These hypotheses are increasingly invoked to explain variations in incidence of childhood cancers but they do not involve ionising radiation. We therefore discuss them in an Appendix.

#### Summary

There is no doubt that there were clusters of LNHL, notably in the vicinity of the Sellafield reprocessing plant, but also around Dounreay and Krümmel. The clusters have been extensively studied. Dose assessments indicate that the clusters do not appear to be due to planned or accidental releases of radioactivity from the plant. Nor does it seem plausible that they are due to occupational exposures of the parents of the children (see section Parental Preconceptional Irradiation (PPI).

### Studies of fallout

One response to the discovery of clusters of childhood leukaemia around Sellafield and Dounreay was the suggestion that the radionuclides released by the plants were very much more dangerous than generally supposed^31^. This could be tested by examining the way that global patterns of incidence of childhood leukaemia varied during the 1950s and early 1960s when large quantities of very similar nuclides were released into the atmosphere as a result of the atmospheric testing of nuclear weapons. The NRCT provided the largest dataset for a study using data from 11 cancer registries in nine countries^32^, which failed to find a discernible increase in childhood leukaemia following the period when the doses were highest.

Apart from fallout from nuclear weapons testing, the largest release of man-made radioactivity into the environment came from the Chernobyl accident^33^. High levels of radioactive iodine were released in the accident and consumption of contaminated milk led to high thyroid doses in parts of Belarus, the Russian Federation and Ukraine^34^ with a clear increase in thyroid cancer in those exposed as children or adolescents^33^. Naturally, the question of possible increases in childhood leukaemia was raised and the NRCT was a contributor to the European Childhood Leukaemia—Lymphoma Incidence Study (ECLIS)^35^. While there was a slight increase in the incidence of childhood leukaemia in Europe after the accident, the overall geographical pattern of change bore no relation to the estimated resultant radiation exposure^35^.

### Other environmental contamination

Dalgety Bay, in Fife, lies on the north bank of the Firth of Forth. After the Second World War, an RAF establishment destroyed unwanted aircraft by incineration, burying them as landfill on the site. It transpired that some of these aircraft had incorporated instruments which had been luminised with paint containing Ra-226. In 1990 a survey of the local beach found discrete sources of this isotope. Further surveys were undertaken, both immediately after the initial discovery and over the following years. Further discoveries of Ra-226 were made. It became apparent that the beach area was undergoing constant remodelling as a result of erosion and redeposition. In consequence, finding and dealing with the contamination is not easy. Both SEPA (the Scottish Environmental Protection Agency) and COMARE have reviewed the situation and made recommendations on how it should be handled. Members of CCRG have been active in the work of COMARE, and in particular in the production of its fifteenth report^36^, which was devoted to the contamination at Dalgety Bay.

In 1996 journalists drew attention to a recently declassified internal AWE report from 1961, which suggested that significant quantities of U-235 had been released into the environment around the US airbase at Greenham Common in Berkshire. This was advanced as a possible cause of increased incidence of childhood leukaemia in the area. Bithell & Draper^37^ re-examined the evidence. They concluded that, although the excess uranium found had a non-random distribution it did not support the pattern depicted in the 1961 report and bore no relation to the incidence of childhood leukaemia. In any case, the increase in level of environmental radiation as a result of the putative release must have been very small.

### Parental preconceptional irradiation

#### The Gardner hypothesis

In 1990, Professor Martin Gardner and colleagues published the results of a case-control study^38^, suggesting that high doses of radiation received by men before they fathered children were associated with LNHL in their offspring. This strong and unexpected association offered a novel potential explanation for the Sellafield cluster which overcame the difficulty that the estimated doses to the children themselves were far too low, on the basis of accepted risk estimates, to account for the observed levels of leukaemia^39^.

A number of investigations were started to explore the Gardner Hypothesis, as it became known. Prominent amongst these were the 'Record Linkage Studies' by CCRG/NRPB. These studies were based on identifying parents of cases from the NRCT and matched controls and ascertaining which were included in the National Registry for Radiation Workers, a large database maintained by NRPB with details of nuclear workers and their occupational exposure to ionising radiation. It was then a matter of comparing the relative numbers of links to the parents of cases and controls and comparing the doses that the respective parents had incurred. This was the first study for which controls were selected for NRCT cases, although some cases and controls were also used from the OSCC^40^ and from the Scottish Study of PPI^41^.

The first of the Record Linkage Studies^42^ found that there was indeed a raised risk of LNHL in the offspring of radiation workers (relative risk 1.77, (1.05–3.03) after exclusion of the Gardner cases but there was no dose-response relation for either of the exposure periods studied. No increased risk was found for fathers with a lifetime preconception dose of 100 mSv or more, or with a dose in the 6 months before conception of 10 mSv or more. Paradoxically, the association was greatest for those who were monitored for exposure to radiation but whose doses were below the level of detection. There was no increased risk for the group of other childhood cancers. The result thus did not support the hypothesis that paternal preconception irradiation is a cause of childhood LNHL. The numbers of mothers who had been radiation workers were very small. However, mothers’ radiation work were associated with an increased risk of childhood cancer overall with a relative risk of 5.00 (1.42–26.94). The statistical uncertainties were large, but this finding was flagged for further investigation when more data were available.

In order to try to explain the apparent paradox relating to fathers’ exposures, Sorahan et al.^43^ undertook a further analysis of what was essentially the same cohort as that studied by Draper et al.^44^. However, the focus was now on the timing of employment at the nuclear site in relation to the conception of the children. The conclusion was that risk was restricted to those working at the site at the time of conception of the child. Men did not carry risk away with them after employment as would be expected if some lasting molecular damage to their germ-cells was involved.

Bunch et al.^45^ studied the mothers of the same cohort together with an additional 16,964 childhood cancer patients taken from the NRCT, together with the same number of matched controls. Pooled analyses, based on the new and original datasets, include 52,612 case and control mothers. The new data provide no evidence of significantly increased risk of childhood cancer with mother’s radiation work, nor was there any increased risk in subgroups that might be taken to be at particular risk (higher dose groups, those monitored for internal exposures or women exposed while pregnant). The investigators concluded that neither the new nor the pooled data support suggestions of increased childhood cancer risks in offspring of female radiation workers.

#### Summary

The 'Gardner hypothesis', that Preconceptional Paternal Irradiation could lead to cancer in the offspring of those exposed, appeared to offer a mechanism that might explain the Sellafield cluster. However, further investigations did not support this hypothesis which has now been largely abandoned. Equally, the parallel hypothesis involving the preconceptional irradiation of mothers has received no support.

### Doses from natural radiation

#### Doses from natural radiation and their predicted consequences

Under virtually all circumstances the greatest doses to populations and individuals come from medical or natural sources. Natural sources of ionising radiation are included in reviews of population exposures eg ref.^7^ but have tended to attract less attention than artificial sources.

Accordingly, a number of studies were carried out to investigate doses from natural sources of radiation, with particular reference to doses to the red bone marrow, thought to be a target tissue for leukaemia induction. These studies included a much more detailed analysis than previously possible of the age-dependent doses to children from natural radiation sources, with assessments at ages 1 year, 5 years, 10 years, 15 years, and adult. Investigations were conducted of

- Doses from radon and decay products to children^46^;
- An overarching review of the variations in radiation exposures of adults and children in the UK^47^;
- Effective and organ doses from the decay products of thoron (Rn-220) to adults and children^48^;
- Doses to the red bone marrow from radiation of natural origin^49,50^.

These age-dependent dose data were used to update estimates of the numbers of childhood leukaemias that might be caused by natural radiation^51–53^. They suggest that natural background radiation causes around 15–20% of all childhood leukaemia cases in Great Britain^54^.

The dosimetric calculations were used in an investigation of the size of epidemiological studies that would be needed in order to demonstrate that natural radiation gives rise to childhood leukaemia^55^. It was concluded that such studies must include ~10,000 cases in order to detect the very small effects expected. Many of the published studies in this area were found to be underpowered, i.e. too small reliably to detect effects of interest.

In addition, work motivated by epidemiology and directed towards estimating individual doses from terrestrial gamma rays has allowed a much better understanding than hitherto of population exposures from this source^56^.

A number of epidemiological studies have investigated whether the very small excess of childhood cancer caused by natural radiation could be detected. These are discussed in the following sections.

#### Ecological Studies of natural background radiation and childhood cancer

Ecological studies, investigating small areas rather than individuals, have methodological disadvantages. In particular, they are potentially subject to the effects of confounding by factors which cannot be allowed for in the analysis. For a fuller discussion see ref.^57^. However, they have practical advantages in terms of speed, size and simplicity. Some ecological investigations of childhood cancer and natural radiation have been carried out using the NRCT.

Muirhead and colleagues conducted a correlation study of rates of LNHL and natural radiation in 459 CDs in England, Scotland and Wales^58,59^. The study included about 6700 cases of LNHL from the NRCT for the period 1969–1983. The radiation data were average indoor and outdoor gamma and indoor radon. For data at County level, the regression coefficient for radon was positive and for both indoor and outdoor gamma it was negative (all significance levels 0.05 < *p* < 0.10). For analyses between Districts within Counties the trend was negative for radon and positive for indoor gamma. For analyses between all CDs, unadjusted for County, none of the regression coefficients differed significantly from zero. Muirhead et al conclude that the difference between the analysis based on counties and that based on districts within counties indicates that the county level analysis is affected by geographical confounding factors.

Richardson et al.^60^ undertook another analysis of essentially the same leukaemia cases and radiation data. Analyses of the geographical variation of childhood leukaemia incidence were carried out using Poisson regressions and also a hierarchical Bayesian model. The main finding was that part of the geographical variation was due to a local neighbourhood clustering structure. It was hypothesized that this might be a consequence of 'a complex combination of environmental and socio-demographic local characteristics'. There was evidence for a positive association of leukaemia incidence with socioeconomic status (SES) score. There was no consistent evidence of a positive association of childhood leukaemia incidence with gamma radiation levels; conversely there was some evidence of an inverse association. There was no consistent evidence of any association with radon levels.

#### Record-based case-control study of natural background radiation and childhood cancer

Conventional interview-based case-control studies of natural radiation and childhood cancer must be impractically large if they are to have sufficient power; moreover, incomplete responses lead to biases which potentially swamp any radiation effects^55^. However, record-based case-control studies make use of pre-existing databases. They do not attempt to make individual contact with the study subjects. Such studies can be very big and they are free of response bias. However, there are no measurements of exposures directly related to study subjects and no data from interviews. In conjunction with other researchers, CCRG has undertaken a study of this kind. The first phase of this study has been published^61,62^. Cases (27,447) were those born and diagnosed in GB during 1980–2006 and were drawn from the National Registry of Childhood Tumours (NRCT). Matched controls (36,793) were also available.

Two sources of information on SES were: Carstairs index of deprivation^63^ calculated for census wards and Father’s Social Class based on occupation as given on the child’s birth certificate.

Two types of radiation exposure were considered. Estimates of mean indoor gamma-ray exposures (with the ionising component of cosmic rays) for 459 CDs were based on a National Survey of indoor exposure to naturally occurring radiation sources^64^. Two measures of indoor radon exposure were available: firstly, means for CDs based on the National Survey (analogues of gamma-ray estimates); secondly, more precise estimates from a predictive map based on domestic measurements grouped by geological boundaries^65^.

For gamma-rays, for the grouping of childhood cancers other than leukaemia the odds ratio was above 1.0, but not close to significance. However, the leukaemia odds ratio, 1.09, was significantly elevated (*p* = 0.01). For radon all odds ratios were above 1.0, but none was close to statistical significance. Broadly similar results were obtained for variants on the main analysis using different measures of SES, different measures of radon exposure and different latent periods

The gamma-ray result is equivalent to a 12% (3–22%; two-sided *p* = 0.01) proportional increase in the risk of childhood leukaemia per millisievert of cumulative red bone marrow dose from gamma radiation; this is broadly compatible with risk estimates of UNSCEAR and BEIR VI^61^. The authors were unable to suggest any confounders that might correlate with exposure to natural background gamma radiation. They regarded the association between childhood leukaemia and naturally occurring gamma-rays as likely to be causal.

### Medical exposures

Researchers, largely from the University of Newcastle and from the National Cancer Institute (USA) found a positive association between radiation dose from CT scans and leukaemia and brain tumours in children and young adults^66^. However, the apparent effects of low-dose radiation might have been influenced by underlying conditions in the patients. CCRG were involved in a detailed investigation of this question which concluded that although there was evidence of some bias in the original risk estimates, re-analysis with additional clinical data, partly from the NRCT, still showed an increased cancer risk after low-dose radiation exposure from CT scans^5^. The bias was somewhat larger in brain tumour cases than for cases with leukaemia/myelodysplastic syndrome.

### Second tumours

The great improvement in treatment of children’s cancer means that more and more patients are surviving for years or can be considered to have been cured. However, this opens up another concern. The therapies for cancers include both radiation and very active chemicals, both of which may be carcinogenic. These second tumours are important both because of the advice and support that should be offered to the patients, but also for the light that they can throw on the risks of such exposures. It is the second of these questions that we consider here.

Of course there are formidable problems in trying to assess the carcinogenic risks of radiation exposure from second tumours in cancer patients. Firstly it is necessary to distinguish between second tumours induced by the treatment from the delayed recognition of cancers traceable to the original disease or its cause. Secondly, the radiotherapy will have been very carefully targeted so as to minimise the dose to non-malignant tissue; the exposures relevant to second tumours will often be due to the fringes of the beam and thus be hard to assess. Thirdly, the effects of radiotherapy must be distinguished from those of chemotherapy.

In the first UK investigation of second tumours in the survivors of childhood cancer, Hawkins et al.^67^, using data from the NRCT, studied 10,106 survivors of a primary childhood cancer. Ninety of these patients were found to have developed a second tumour that was diagnosed more than three years after the primary was diagnosed. Second tumours diagnosed within three years of the primary were excluded because in this period recurrence and metastatic spread of the primary are most common. The risks of a second tumour were clearly elevated over those expected in the general population and were higher in those who had received radiotherapy. Within 25 years of three year survival about 4% developed a second primary tumour, about six times the expected frequency. Hawkins et al.^68^ later extended this cohort.

In a study of secondary leukaemia after childhood cancer, Hawkins et al.^69^ noted that the relative risk of secondary leukaemia increased significantly with dose of radiation averaged over patients’ active bone marrow. These populations were also used by Little and colleagues in investigations^70,71^ of the way that risk of various types of cancer varies after exposure. Little et al. used data from the atomic bomb survivors and five cohorts exposed in childhood for medical reasons to derive a preferred relative risk model with adjustments to the excess relative risk proportional to a product of powers of time since exposure and attained age. This allowed solid cancer incidence and mortality risks to be estimated for the UK population.

The cancer survivor database moved to the University of Birmingham in 2000 and is known as the British Childhood Cancer Survivor Study^72^.

## Discussion

Immediately after its foundation in 1975, the work of the CCRG largely involved matters other than ionising radiation and indeed was not concentrating specifically on aetiological epidemiology^2^. However, this changed with the reporting of the Sellafield cluster in 1983 and of another near Dounreay in 1986. The CCRG was involved in the work of the Black Committee^10^ and deeply involved with its successor COMARE. During the period in question, COMARE was an influential and high-profile government committee^73^, frequently cited in Hansard. COMARE leaned heavily on the data and expertise of the CCRG from its inception to the group’s closure in 2014 and this involvement accounted for a large fraction of the Group’s work. The effect of the closure on the work of COMARE has already been noted^74^.

Confirmation of the existence of clusters of LNHL at Sellafield and Dounreay and the understanding that ionising radiation did not offer a plausible explanation would have been impossible without the contribution from CCRG. The role of the CCRG in investigating parental preconceptional irradiation was particularly relevant.

Fallout from nuclear weapons and from nuclear accidents has naturally aroused public concern and CCRG work has been important in investigating these topics.

Work by CCRG has increased our knowledge of the doses incurred by members of the public from natural radiation. It has also included an epidemiological study of natural radiation and childhood cancer which was large enough to detect the very small expected effect and to support the contention that radiation effects continue down to very low doses.

Second tumours in survivors of childhood cancer are of great importance in their own right. In the present context they are also important for the light that they throw on the induction of such second tumours by radiotherapy for the initial disease.

Over the 40 years of its existence, the CCRG accumulated considerable experience and analytical expertise which, together with the unique data resource represented by the NRCT, have contributed greatly to advances in understanding of the association between exposure to ionising radiation and subsequent cancer.

## Appendix

**Infective mechanisms and clusters of childhood leukaemia**

Two main hypotheses concerning infectious mechanisms in the aetiology of childhood leukaemia have been advanced: Greaves’s Delayed Infection hypothesis^75^ and Kinlen’s Population Mixing Hypothesis^76^. The Population Mixing Hypothesis has been extended by other researchers. In addition it has been suggested by workers from CCRG that under-diagnosis of childhood leukaemia, varying both with calendar period and with SES might play a role.

The steady increase in support for these infection-based hypotheses and the dwindling of support for alternatives has left them as widely regarded as the most plausible explanations for clustering and as responsible for many features of childhood leukaemia. However, the available evidence does not allow a clear choice between them. McNally and Eden reviewed the evidence^77^ and concluded 'It is very important to realize that the Greaves (1988) and Kinlen population mixing hypotheses are not mutually exclusive. Elements of both may be involved in individual cases.'

For more recent reviews of clustering see ref.^2,78^.

**Greaves’s delayed infection hypothesis**

Greaves suggested that precursor B-cell Acute Lymphoblastic Leukaemia might require two independent mutations^75,79^. Infection was postulated to have a crucial role in promoting, through the immune response, the crucial second or postnatal genetic error. Greaves pointed out that absence or diminution of infections early in life is a feature of more affluent ‘hygienic’ societies. This has produced substantial benefits in terms of reduced infant mortality. However, such infectious insulation might predispose the immune system to aberrant or pathological responses following subsequent or ‘delayed’ exposure.

Greaves and Alexander^80^ published a comprehensive review of theories of an infectious aetiology for childhood leukaemia. The Greaves hypothesis receives support from, for example, an investigation from the UKCCS into day care in infancy and the risk of ALL^81^ which showed that increasing levels of social activity were associated with consistent reductions in risk of ALL.

**Kinlen’s population mixing hypothesis**

A possible explanation for clusters of childhood leukaemia around nuclear sites (and elsewhere) has been suggested by Kinlen^76,82^ who proposed a population mixing hypothesis under which

- Some childhood leukaemia is a rare response to an as yet unidentified infection;
- Individuals in isolated or rural areas would be less likely to have been exposed to this agent in early life and would be susceptible to infection by it later;
- Marked influxes of people into rural areas would lead to mini-epidemics of subclinical infections by this agent; such infections are mainly immunizing but in rare cases lead to childhood leukaemia.

This hypothesis does not involve ionising radiation but it is frequently discussed in the context of clusters. Several studies have been published that support this idea^82^ and it has been gaining acceptance^83^. The NRCT has provided data to test it^84^.

**Extended population mixing hypothesis**

Some studies have considered population mixing in a broader sense than that defined by Kinlen^82^. In Kinlen’s sense, population mixing requires striking increases of population in rural areas. These other studies examine childhood leukaemia rates in the context of variables such as immigration rates in areas where there is no such dramatic influx into an isolated rural community.

Thus Stiller and co-workers^85,86^ using NRCT data found increased levels of childhood leukaemia in areas with greater levels of population influx both for CDs and for Census wards. Dickinson and co-workers, using data partly from the NRCT, found elevated levels of childhood ALL in electoral wards with the highest levels of population mixing^87^. They applied these ideas to studies of cancers (particularly LNHL) in the children of Cumbrian nuclear workers where measures of population mixing were again associated with childhood cancer. CCRG staff were again involved in some of this work^88^.

**Pre-emptive infection**

Another way in which infection might affect childhood cancer rates, possibly including the Sellafield cluster, is that ‘pre-emptive infection’ might be a mechanism explaining increasing time trends in recorded childhood acute lymphoblastic leukaemia incidence, and relatively low rates in children from more deprived communities^89–92^. Under this hypothesis, acute leukaemia in children pre-disposes to fatal infection, and does not always have obvious clinical symptoms. Some children might die of such infections without leukaemia ever being diagnosed. In Britain, this would probably have been more frequent in the 1970s and 80s and in more deprived communities. Clinical evidence from the 1980s and 90s supports this suggestion in the context of the socioeconomic gradient^93^. Greater awareness of the possibility of cancer around nuclear installations might have resulted in a smaller chance of leukaemias being missed than in other areas and under-diagnosis is likely to have been greater in the 1960s. However, it is highly implausible that such an effect could be large enough to explain the Sellafield cluster fully.
